# Coronavirus-associated kidney outcomes in COVID-19, SARS, and MERS: a meta-analysis and systematic review

**DOI:** 10.1080/0886022X.2020.1847724

**Published:** 2020-11-30

**Authors:** Shoulian Zhou, Jing Xu, Cheng Xue, Bo Yang, Zhiguo Mao, Albert C. M. Ong

**Affiliations:** a Division of Nephrology, Changzheng Hospital, Second Military Medical University, Shanghai, People’s Republic of China; b Internal Medicine III (Nephrology & Endocrinology), Naval Medical Center of PLA, Second Military Medical University, Shanghai, People’s Republic of China; c Academic Nephrology Unit, Department of Infection, Immunity and Cardiovascular Disease, University of Sheffield, Sheffield, UK; d Sheffield Teaching Hospitals NHS Trust, Sheffield Kidney Institute, Sheffield, UK

**Keywords:** COVID-19, SARS, MERS, kidney, outcome

## Abstract

**Objectives:**

A meta-analysis and systematic review was conducted on kidney-related outcomes of three recent pandemics: SARS, MERS, and COVID-19, which were associated with potentially fatal acute respiratory distress syndrome (ARDS).

**Methods:**

A search of all published studies until 16 June 2020 was performed. The incidence/prevalence and mortality risk of acute and chronic renal events were evaluated, virus prevalence, and mortality in preexisting hemodialysis patients was investigated.

**Results:**

A total of 58 eligible studies involving 13452 hospitalized patients with three types of coronavirus infection were included. The reported incidence of new-onset acute kidney injury (AKI) was 12.5% (95% CI: 7.6%–18.3%). AKI significantly increased the mortality risk (OR = 5.75, 95% CI 3.75–8.77, *p* < 0.00001) in patients with coronavirus infection. The overall rate of urgent-start kidney replacement therapy (urgent-start KRT) use was 8.9% (95% CI: 5.0%–13.8%) and those who received urgent-start KRT had a higher risk of mortality (OR = 3.43, 95% CI 2.02–5.85, *p* < 0.00001). Patients with known chronic kidney disease (CKD) had a higher mortality than those without CKD (OR = 1.97, 95% CI 1.56–2.49, *p* < 0.00001). The incidence of coronavirus infection was 7.7% (95% CI: 4.9%–11.1%) in prevalent hemodialysis patients with an overall mortality rate of 26.2% (95% CI: 20.6%–32.6%).

**Conclusions:**

Primary kidney involvement is common with coronavirus infection and is associated with significantly increased mortality. The recognition of AKI, CKD, and urgent-start KRT as major risk factors for mortality in coronavirus-infected patients are important steps in reducing future mortality and long-term morbidity in hospitalized patients with coronavirus infection.

## Introduction

The novel coronavirus disease-2019 (COVID-19), first reported in Wuhan, China, has become a worldwide pandemic and has caused over 28,918,900 confirmed cases of COVID-19 globally, including 922,252 deaths reported to the World Health Organization (WHO) as of 3:28 pm CEST, 14 September 2020 [1]. Apart from the rapid development of acute respiratory distress syndrome (ARDS), severe acute respiratory syndrome coronavirus 2 (SARS-CoV-2), as well as the previously identified severe acute respiratory syndrome coronavirus (SARS-CoV-1) and Middle East respiratory syndrome coronavirus (MERS-CoV), which are members of the coronavirus family [2], also have major associated but under-recognized extrapulmonary manifestations [3–5]. These three types of coronaviruses have caused catastrophic coronavirus pandemics in human history, namely SARS, MERS, and COVID-19. Among the organs affected, the kidneys are often involved due to the organ cross-talk between alveolar and tubular damage, i.e. the lung–kidney axis in ARDS [6]; the occurrence of kidney involvement usually indicates a worse prognosis [7,8]. Although the etiology of coronavirus-associated AKI is likely to be multifactorial, all three coronaviruses can directly invade renal cells through hijacking native surface receptors: angiotensin-converting enzyme 2 (ACE2) serves as a receptor for SARS-CoV-1 and SARS-CoV-2 [9,10], while MERS-CoV enters target cells *via* binding to dipeptidyl-peptidase 4 (DDP4) [11]. However, it is unclear how the virus causes cellular damage following the entry. If maintained during the course of infection, the kidney could function as a viral reservoir and urine become a potential source of viral transmission.

Acute kidney injury (AKI) is the most frequent extra-pulmonary organ dysfunction associated with ARDS and is an independent risk factor for mortality [12,13]. However, the reported prevalence and mortality of AKI for all three coronavirus infection differs between studies. All patients with chronic kidney disease (CKD), including those with end-stage kidney disease (ESKD) or on kidney replacement therapy (KRT), are immunosuppressed making them more susceptible to infection and potentially a more severe course [14–16]. The potential increased risk related to preexisting CKD and urgent-start KRT treatment is presently unclear. The influence of SARS-CoV-2/COVID-19 on sustained dialysis patients is also unknown.

In view of our previous experience with SARS, MERS, and more recent experiences of the COVID-19 outbreaks, we conducted a meta-analysis and systematic review to investigate the kidney involvement and patients’ outcomes in hospitalized coronavirus-infected patients.

## Methods

### Data sources and search

This systematic review was performed following PRISMA (Preferred Reporting Items for Systematic Reviews and Meta-Analyses) guidelines. The registration of this review was published in PROSPERO (CRD42020200941). A search for published studies was performed using the PubMed database, EMBASE, and Cochrane library until 16 June 2020. Research articles on coronavirus (SARS-CoV, MERS-CoV, and SARS-CoV2) infected patients with information on kidney disease, AKI, dialysis, or kidney function were eligible and included. Keywords (‘COVID-19′ OR ‘SARS-CoV2’) or ‘SARS-CoV’ or ‘MERS-CoV’ and (‘chronic kidney disease’ or ‘CKD’ or ‘kidney disease’ or ‘end-stage kidney disease’ or ‘ESKD’) or (‘acute kidney injury’ or ‘AKI’) or (‘kidney replacement therapy’ or ‘KRT’ or ‘blood purification’) or (‘dialysis’ or ‘hemodialysis’ or ‘blood purification’) or (‘mortality’ or ‘death’) were combined to construct corresponding search formulas in databases. We used a combination of subject terms with free-text terms during the search, supplemented by a manual search and citation search. We also screened the latest relevant articles about COVID-2019 and met the inclusion criteria, through the "https://www.biorxiv.org/search/covid-19" website.

### Inclusion and exclusion criteria

Studies that met the following PICOS criteria were included: (1) articles were original reports including patients infected with coronavirus; (2) studies with outcomes of interest consisting of mortality or kidney-related outcomes, i.e. urgent-start KRT, AKI or dialysis; (3) types of articles were cohort studies, case series, and case-control studies. The exclusion criteria of the study included articles reporting patients infected with coronavirus other than SARS-CoV, MERS-CoV, and SARS-CoV2; articles without relevant outcomes of interest; commentaries or reviews; research articles with patient numbers below five.

### Study selection and data extraction

Two reviewers (ZS and XC) independently screened the titles and abstracts and then checked the full text of all the articles that might be eligible. Differences were resolved through discussion or consultation with the third reviewer (XJ). The two researchers separately extracted data from the included studies, including first author of the article, year, study design, follow-up, number of reported cases, mortality, CKD, AKI, use of urgent-start KRT, ESKD, incidence or mortality of infected dialysis patients and related baseline characteristics. Mortality was defined as the death of patients during hospitalization.

The quality rating for each study was evaluated by the NOS (Newcastle-Ottawa scale). For the evaluation of case reports and case series included, we applied a generally recommended standard similar to NOS, based on the domains of selection, ascertainment, causality and reporting and provide signaling questions [17].

### Patient and public involvement

No patient involved.

### Certainty rating of evidence

The GRADE instrument (Grading of Recommendation, Assessment, Development, and Evaluation) was applied to rate the certainty of evidence and the strength of recommendations generated in our study [18–20]. The certainty of evidence was rated for kidney-related complications and underlying kidney diseases prevalence and associations with patients’ prognosis. The five GRADE considerations (study limitations, consistency of effect, imprecision, indirectness and publication bias) were taken into account to assess the confidence in effect estimates. Quality of evidence was characterized as high, moderate, low, or very low [20,21]. GRADE was assessed per http://gradepro.org.

### Statistical analysis

Assessment of risk of bias was performed by two authors (XC and XJ) independently using the Newcastle–Ottawa Quality Assessment Scale (NOS) [22]. Studies were scored up to a maximum of 9 points by NOS. Study quality was classified into three categories: 0–3 (low), 4–6 (moderate), and 7–9 (high). Statistical analyses were performed using Revman software V5.4 (Cochrane). Sensitivity analysis on the results of pooled analysis was performed by method of excluding all the included preprinted literatures and method of one-by-one elimination to verify the stability of the results. Rates for dichotomous data were analyzed using the Stata 16.0 (Stata Corp LP, TX, USA) Metaprop package. Since most studies were of retrospective design and heterogeneity between studies expected, the random-effects model was chosen for data synthesis [23]. Odds Ratio (ORs) and 95% confidence intervals (CIs) were used for dichotomous variables as effect measures and were graphically visualized using Forest plots. Besides, *T*-statistic using Hartung–Knapp–Sidik–Jonkman method was performed for the degrees of freedom in the random-effects analysis, when the number of studies was < 10 by R version 4.03 (Metafor package) [24]. Heterogeneity across studies was evaluated using the Cochrane *Q* test and *I*^2^ test (*I*^2^ = 100% ((Q-df)/Q). An I^2^ value of 0–49%, 50–74% and >75% indicated low, moderate or high heterogeneity, respectively [25]. A two-sided P value < 0.10 was considered statistically significant. Subgroup analysis was performed for each individual virus. If the number of studies was <9, publication bias was not investigated. Publication bias was evaluated with Begg’s test, Egger’s test and Funnel-plot. Meta-regression analysis was used to find potential heterogeneity. A two-sided *P*-value <0.05 except heterogeneity was considered statistically significant.

## Results

### Search results and study selection

**Figure 1.**
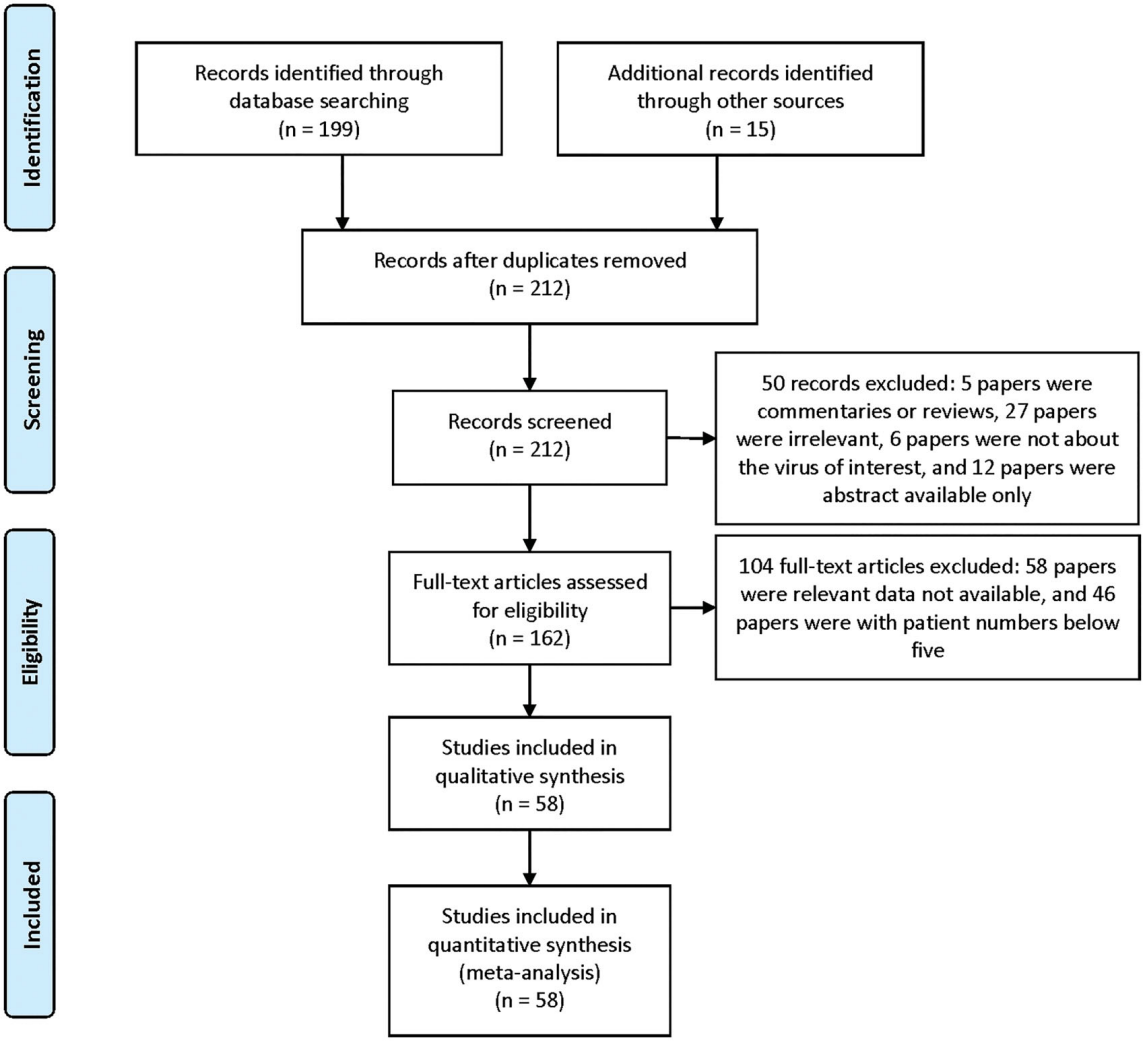
Flow chart of the diagram. SARS: severe acute respiratory syndrome; MERS: Middle East respiratory syndrome; COVID-19: novel coronavirus disease 2019.

Our initial search strategy identified 42 papers on SARS, 32 papers on MERS and 125 papers on COVID-19 (199 records) displayed in Figure 1. The search strategy was listed in Supplementary 1. A further search was then performed on citation and online preprint servers identifying 15 further papers, resulting in a total of 214 records. Following a thorough assessment, 58 articles with 13452 patients were included for quantitative synthesis and further investigation (Tables 1 and 2). Most COVID-19 studies were performed in Asia (68%, *n* = 21) [6,26–45], followed by Europe (19%, *n* = 6) [46–51] and North America (13%, *n* = 4) [52–55]. Most SARS studies were performed in Asia (88%, *n* = 7) [5,56–61] except for one study from Canada[62], and almost all the MERS studies were performed in Saudi Arabia (95%, *n* = 18) [3,63–79] with the exception of one study from South Korea. Follow-up duration ranged from 1 to 46 months. The publication years of these studies ranged from 2003 to 2020. All studies on COVID-19 were published within the first 6 months of 2020.

**Table 1.** Summary of the characteristics of the enrolled studies.

Author year	Study type	Types of diseases infected	Outbreak site	Time (months)	Total death (n)	Total cases (n)	Age (mean ± SD or IQRs)	AKI incidence (n)	AKI mortality (n)	CKD patients (n)	CKD mortality (n)
Wong 2003	Retrospective cohort study	SARS	Hong Kong, China	NA	6	15	66.3 ± 13.5 years	1/15 (6.67%)	1/1 (100.00%)	4/15 (26.67%)	4/4 (100.00%)
Gu 2005	Case series	SARS	Beijing, China	NA	8	8	41.9 ± 14.5 years	NA	NA	0/8 (0%)	NA
Chen 2005	Retrospective cohort study	SARS	Taiwan, China	2	4	30	40 years (range, 12–87 years)	3/30 (10.00%)	2/3 (66.67%)	NA	NA
Wu 2005	Case-control study	SARS	Taiwan, China	1	14	60	47 ± 17 years	11/60 (18.33%)	NA	0/60 (0%)	NA
Farcas 2005	Retrospective cohort study	SARS	Toronto, Canada	6	19	19	68.8 ± 15.5 years	NA	NA	0/19 (0%)	NA
Chu 2005	Retrospective cohort study	SARS	Hong Kong, China	NA	33	536	53.5 years (range, 34–77 years)	36/536 (6.71%)	33/36 (91.67%)	0/536 (0%)	NA
Kwan 2004	Retrospective cohort study	SARS	Hong Kong, China	4	9	35	58 years (range, 34–74 years)	NA	NA	12/35 (34.29%)	3/12 (25.00%)
Peiris 2003	Prospective cohort study	SARS	Hong Kong, China	<1	5	75	39.8 ± 12.2 years	NA	NA	NA	NA
Al-Jasser 2019	Retrospective cohort study	MERS	Saudi Arabia	20	50	249	46.71 ± 17.92 years	NA	NA	29/249 (11.25%)	NA
Assiri 2016	Retrospective cohort study	MERS	Saudi Arabia	6	21	38	51 years (range, 17–84 years)	NA	NA	12/38 (31.58%)	9/12 (75.00%)
Garout 2018	Retrospective cohort study	MERS	Saudi Arabia	4	39	52	NA	NA	NA	11/52 (21.15%)	11/11 (100.00%)
Sherbini 2017	Retrospective cohort study	MERS	Saudi Arabia	2	10	29	45 years (range, NA)	NA	NA	8/21 (27.59%)	8/8 (100.00%）
Shalhoub 2015	Retrospective cohort study	MERS	Saudi Arabia	1	22	32	NA	NA	NA	14/32 (43.75%)	14/14 (100.00%）
Hastings 2016	Retrospective cohort study	MERS	Saudi Arabia	2	NA	78	53 years (range, NA)	NA	NA	11/78 (14.10%)	NA
Arabi 2017	Retrospective cohort study	MERS	Saudi Arabia	37	217	330	58 years (range, 44–69 years)	NA	NA	100/330 (30.30%)	80/100 (80.00%)
Assiri 2013	Retrospective cohort study	MERS	Saudi Arabia	9	28	47	NA	NA	NA	23/47 (48.93%)	17/23 (73.91%)
Alfaraj 2019 (1)	Case series	MERS	Saudi Arabia	46	0	7	8.04 ± 5.67 years	NA	NA	1/7 (14.29%)	0/1 (0%）
Alqahtani 2018	Retrospective cohort study	MERS	Saudi Arabia	26	55	281	NA	NA	NA	9/281 (3.20%)	6/9 (66.67%)
Alanazi 2019	Retrospective cohort study	MERS	Saudi Arabia	1	13	48	58 years (range, 29–84 years)	NA	NA	10/48 (20.83%)	NA
Alfaraj 2019 (2)	Retrospective cohort study	MERS	Saudi Arabia	47	78	314	48 ± 17.3 years	NA	NA	NA	NA
Al-Tawfiq 2014	Case-Control study	MERS	Saudi Arabia	2	13	17	62 years (range, 14–87 years)	NA	NA	5/17 (29.41%)	NA
Arabi 2014	Case series	MERS	Saudi Arabia	8	7	12	59 years (range, 36–83 years)	7/12 (58.33%)	7/7 (100%)	8/12 (66.67%)	NA
Cha 2015	Retrospective cohort study	MERS	South Korea	2	5	30	54 ± 20.7 years	8/30 (26.67%)	NA	3/30 (10.00%)	NA
Ghamdi 2016	Retrospective cohort study	MERS	Saudi Arabia	11	19	51	54 years (IQR 36.5–58)	NA	NA	14/51 (27.45%)	8/14 (57.14%)
Khalid I 2016	Retrospective cohort study	MERS	Saudi Arabia	1	9	14	54 years (range, 23–79 years)	5/14 (35.71%)	NA	6/14 (42.85%)	3/6 (50%)
Khalid M 2014	Case series	MERS	Saudi Arabia	NA	3	6	58.8 ± 24.7 years	3/6 (50.00%)	3/3 (100%)	0/6 (0%)	NA
Omrani 2014	Retrospective cohort study	MERS	Saudi Arabia	19	34	44	NA	22/44 (50.00%)	NA	11/44 (25.00%)	NA
Ma 2020	Retrospective cohort study	COVID-19	Wuhan, China	1	6	41	66 years (IQR 55–81)	NA	NA	37/41 (90.24%)	6/37 (16.22%)
Richardson 2020	Retrospective cohort study	COVID-19	New York, America	1	553	5700	63 years (IQR 52–75)	1307/5700 (22.93%)	347/1307 (66.35%)	454/5700 (7.96%)	NA
Chen N 2020	Retrospective cohort study	COVID-19	Wuhan, China	<1	11	99	55.5 ± 13.1 years	3/99 (3.03%)	NA	0/99 (0%)	NA
Lu 2020	Retrospective cohort study	COVID-19	Shanghai, China	1	1	265	NA	1/265 (0.37%)	NA	5/265 (1.89%)	NA
Wang D 2020	Retrospective cohort study	COVID-19	Wuhan, China	<1	6	138	56 years (IQR 42–68)	5/138 (3.62%)	NA	4/138 (2.89%)	NA
Chen T 2020	Retrospective cohort study	COVID-19	Wuhan, China	1	113	274	62 years (IQR 44–70)	29/274 (10.58%)	28/29 (96.55%)	4/274 (1.46%)	4/5 (80%)
Xu 2020	Retrospective cohort study	COVID-19	Zhejiang, China	<1	0	62	41 years (IQR 32–52)	NA	NA	1/62 (1.61%)	NA
Huang 2020	Retrospective cohort study	COVID-19	Wuhan, China	1	6	41	49 years (IQR 41–58)	3/41 (21.42%)	NA	0/41 (0%)	NA
Diao 2020	Retrospective cohort study	COVID-19	Wuhan, China	2	NA	85	NA	23/85 (27.06%)	NA	5/85 (5.88%)	NA
Cao 2020	Retrospective cohort study	COVID-19	Wuhan, China	1	17	102	54 years (IQR 37–67)	20/102 (19.61%)	15/20 (75.00%)	4/102 (3.92%)	3/4 (75.00%)
Arentz 2020	Retrospective cohort study	COVID-19	Washington, America	1	11	21	70 years (range, 43–92 years)	4/21 (19.04%)	NA	12/21 (57.14%)	NA
Cheng 2020	Prospective cohort study	COVID-19	Wuhan, China	1	113	701	63 years (IQR 50–71)	36/701 (5.14%)	16/35 (45.71%)	14/698 (2.01%)	NA
Guan 2020	Retrospective cohort study	COVID-19	China	1	15	1099	47 years (IQR 35–58)	6/1099 (0.55%)	NA	8/1099 (0.73%)	NA
Shi 2020	Retrospective cohort study	COVID-19	Wuhan, China	1	57	416	64 years (range, 21–95 years)	8/416 (1.92%)	NA	14/416 (3.36%)	NA
Wang L 2020	Retrospective cohort study	COVID-19	Wuhan, China	1	7	116	54 years (IQR 38–69)	0/116 (0%)	NA	5/116 (4.31%)	0/5 (0%)
Yang 2020	Retrospective cohort study	COVID-19	Wuhan, China	1	32	52	59.7 ± 13.3 years	15/52 (28.85%)	12/15 (80.00%)	0/52 (0%)	NA
Pei 2020	Retrospective cohort study	COVID-19	Wuhan, China	1	29	333	56.3 ± 13.4 years	35/333 (10.51%)	20/35 (57.14%)	NA	NA
Xiong 2020	Retrospective cohort study	COVID-19	Wuhan, China	2	41	131	63.3 ± 13.2 years	NA	NA	NA	NA
Luo 2020	Retrospective cohort study	COVID-19	Wuhan, China	1	100	403	56 years (IQR 39–68)	57/403 (14.14%)	43/57 (75.44%)	7/403 (1.74%)	3/7 (42.86%)
Zhou 2020	Retrospective cohort study	COVID-19	Wuhan, China	1	54	191	56 years (IQR 46–67)	28/191 (14.66%)	27/28 (96.43%)	2/191 (1.05%)	2/2 (100%)
Albalate 2020	Prospective cohort study	COVID-19	Madrid, Spain	1	6	37	NA	NA	NA	NA	NA
Valeri 2020	Retrospective cohort study	COVID-19	New York, America	1	18	59	63 years (IQR 56–78)	NA	NA	NA	NA
Chen M 2020	Retrospective cohort study	COVID-19	Wuhan, China	1	31	123	NA	18/123 (14.63%)	15/18 (83.33%)	7/123 (5.69%)	2/7 (28.57%)
Jung 2020	Retrospective cohort study	COVID-19	South Korea	2	2	14	63.5 ± 14.5 years	NA	NA	NA	NA
Arslan 2020	Case series	COVID-19	Turkey	NA	0	7	64 years (IQR 18–93)	NA	NA	NA	NA
Alberici 2020	Retrospective cohort study	COVID-19	Brescia, Italy	1	27	94	72 years (IQR 62–79)	NA	NA	NA	NA
Goicoechea 2020	Retrospective cohort study	COVID-19	Madrid, Spain	1	11	36	71 ± 12 years	NA	NA	NA	NA
Dudreuilh 2020	Retrospective cohort study	COVID-19	London, UK	NA	NA	34	NA	NA	NA	NA	NA
Trujillo 2020	Retrospective cohort study	COVID-19	Madrid, Spain	NA	13	51	64 ± 15 years	NA	NA	51/51 (100%)	13/51 (25.49%)
Manganaro 2020	Retrospective cohort study	COVID-19	Piedmont and Aosta Valley, Italy	1	39	156	69.7 years (IQR 26–92)	NA	NA	130/156 (83.33%)	NA
Fisher 2020	Retrospective cohort study	COVID-19	New York, America	1	32	114	64.5 years (IQR 55–73)	NA	NA	NA	NA

**Table 2.** Summary of the characteristics of the enrolled studies in patients with ESKD.

Author year	Types	ESKD (n (%))	ESKD mortality (n)	Rate of urgent-start KRT use (n)	urgent-start KRT mortality (n)	Infection rate of HD (n)	Infection mortality in HD patients (n)
Wong 2003	SARS	4/15 (26.67%) (3 PD; 1 HD)	4/4 (100.00%) (PD:3; HD:1)	NA	NA	NA	NA
Gu 2005	SARS	0/8 (0%)	NA	NA	NA	NA	NA
Chen 2005	SARS	NA	NA	NA	NA	NA	NA
Wu 2005	SARS	0/60 (0%)	NA	NA	NA	NA	NA
Farcas 2005	SARS	0/19 (0%)	NA	NA	NA	NA	NA
Chu 2005	SARS	0/536 (0%)	NA	10/536 (1.87%)	NA	NA	NA
Kwan 2004	SARS	12/35 (34.29%) (8 PD; 4 HD)	3/12 (25.00%)	NA	NA	12/700 (1.71%)	3/12 (25.00%)
Peiris 2003	SARS	NA	NA	NA	NA	NA	NA
Al-Jasser 2019	MERS	NA	NA	33/249 (13.25%)	25/33 (75.76%)	NA	NA
Assiri 2016	MERS	12/38 (31.58%) (dialysis:12)	9/12 (75.00%)	NA	NA	12/377 (3.18%)	9/12 (75.00%)
Garout 2018	MERS	NA	NA	NA	NA	NA	NA
Sherbini 2017	MERS	NA	NA	NA	NA	NA	NA
Shalhoub 2015	MERS	8/32 (25.00%)	8/8 (100.00%）	NA	NA	NA	NA
Hastings 2016	MERS	11/78 (14.10%)	NA	NA	NA	11/22 (50.00%)	NA
Arabi 2017	MERS	NA	NA	161/330 (48.79%)	131/161 (81.37%)	NA	NA
Assiri 2013	MERS	NA	NA	NA	NA	NA	NA
Alfaraj 2019 (1)	MERS	1/7 (14.29%)	0/1 (0%）	NA	NA	NA	NA
Alqahtani 2018	MERS	7/281 (2.49%)	4/7 (57.14%)	NA	NA	NA	NA
Alanazi 2019	MERS	NA	NA	NA	NA	NA	NA
Alfaraj 2019 (2)	MERS	NA	NA	NA	NA	NA	NA
Al-Tawfiq 2014	MERS	5/17 (29.41%) (dailysis:5)	NA	NA	NA	NA	NA
Arabi 2014	MERS	2/12 (16.67%) (kidney transplant:1; dialysis:1)	NA	7/12 (58.33%)	NA	NA	NA
Cha 2015	MERS	NA	NA	3/30 (10.00%)	3/3 (100%)	NA	NA
Ghamdi 2016	MERS	14/51 (27.45%)	8/14 (57.14%)	NA	NA	NA	NA
Khalid I 2016	MERS	3/14 (21.43%) (HD:3)	3/3 (100.00%)	5/14 (35.71%)	NA	NA	NA
Khalid M 2014	MERS	0/6 (0%)	NA	3/6 (50.00%)	3/3 (100%)	NA	NA
Omrani 2014	MERS	0/44 (0%)	NA	22/44 (50.00%)	NA	NA	NA
Ma 2020	COVID-19	37/41 (90.24%)	6/37 (16.22%)	NA	NA	37/230 (16.09)	6/37 (16.22%)
Richardson 2020	COVID-19	186/5700 (3.26%)	NA	225/5700 (3.95%）	78/225 (96.30%)	NA	NA
Chen N 2020	COVID-19	0/99 (0%)	NA	9/99 (9.09%)	NA	NA	NA
Lu 2020	COVID-19	NA	NA	2/265 (0.75%)	NA	NA	NA
Wang D 2020	COVID-19	NA	NA	2/138 (1.45%)	NA	NA	NA
Chen T 2020	COVID-19	NA	NA	3/274 (1.09%)	3/3 (100%)	NA	NA
Xu 2020	COVID-19	NA	NA	NA	NA	NA	NA
Huang 2020	COVID-19	NA	NA	3/41 (21.42%)	NA	NA	NA
Diao 2020	COVID-19	NA	NA	NA	NA	NA	NA
Cao 2020	COVID-19	NA	NA	6/102 (5.88%)	5/6 (83.33%)	NA	NA
Arentz 2020	COVID-19	2/21 (9.52%)	NA	NA	NA	NA	NA
Cheng 2020	COVID-19	NA	NA	NA	NA	NA	NA
Guan 2020	COVID-19	NA	NA	9/1099 (0.82%)	NA	NA	NA
Shi 2020	COVID-19	NA	NA	2/416 (0.48%)	NA	NA	NA
Wang L 2020	COVID-19	5/116 (4.31%)	0/5 (0%)	NA	NA	NA	NA
Yang 2020	COVID-19	0/52 (0%)	NA	9/52 (17.31%)	8/9 (88.89%)	NA	NA
Pei 2020	COVID-19	NA	NA	6/333 (17.14%)	NA	NA	NA
Xiong 2020	COVID-19	NA	NA	NA	NA	154/7154 (2.15%)	41/131 (31.30%)
Luo 2020	COVID-19	NA	NA	39/403 (9.68%)	16/39 (41.03%)	NA	NA
Zhou 2020	COVID-19	NA	NA	10/191 (5.24%)	10/10 (100%）	NA	NA
Albalate 2020	COVID-19	NA	NA	NA	NA	37/90 (41.11%)	6/37 (16.22%)
Valeri 2020	COVID-19	NA	NA	NA	NA	NA	18/59 (30.51%)
Chen M 2020	COVID-19	NA	NA	NA	NA	NA	NA
Jung 2020	COVID-19	NA	NA	NA	NA	14/582 (2.41%)	2/14 (14.29%)
Arslan 2020	COVID-19	NA	NA	NA	NA	7/602 (1.16%)	0/7 (0%)
Alberici 2020	COVID-19	NA	NA	NA	NA	94/643 (14.62%)	27/94 (28.72%)
Goicoechea 2020	COVID-19	NA	NA	NA	NA	36/282 (12.77%)	11/36 (30.56%)
Dudreuilh 2020	COVID-19	NA	NA	NA	NA	34/664 (5.12%)	NA
Trujillo 2020	COVID-19	51/51 (100%)	13/51 (25.49%)	NA	NA	NA	7/25 (28.00%)
Manganaro 2020	COVID-19	130/156 (83.33)	NA	NA	NA	102/3280 (3.11%)	NA
Fisher 2020	COVID-19	NA	NA	NA	NA	NA	32/114 (28.07%)

### Quality rating

The quality rating for each study was evaluated by the NOS (Newcastle-Ottawa scale) (Supplementary Table 1). The average score of the included studies was 5.6 indicating moderate quality. The average score of studies for SARS, MERS, and COVID-19 were 5.8, 6.2, and 5.2, respectively. In domains of comparability, 52 studies with single arms did not fulfill the selection of the non-exposed cohort and only six studies [43,45,59,61,71,80] received scores. The GRADE tool was used to summarize pooled evidence for the main outcomes, as shown in Supplementary Table 2. Except several subgroup comparisons were rated as low, the majority of recommendations generated from this systematic review and meta-analysis were evaluated as very low.

### AKI incidence and mortality risk in patients with coronavirus infection

**Figure 2.**
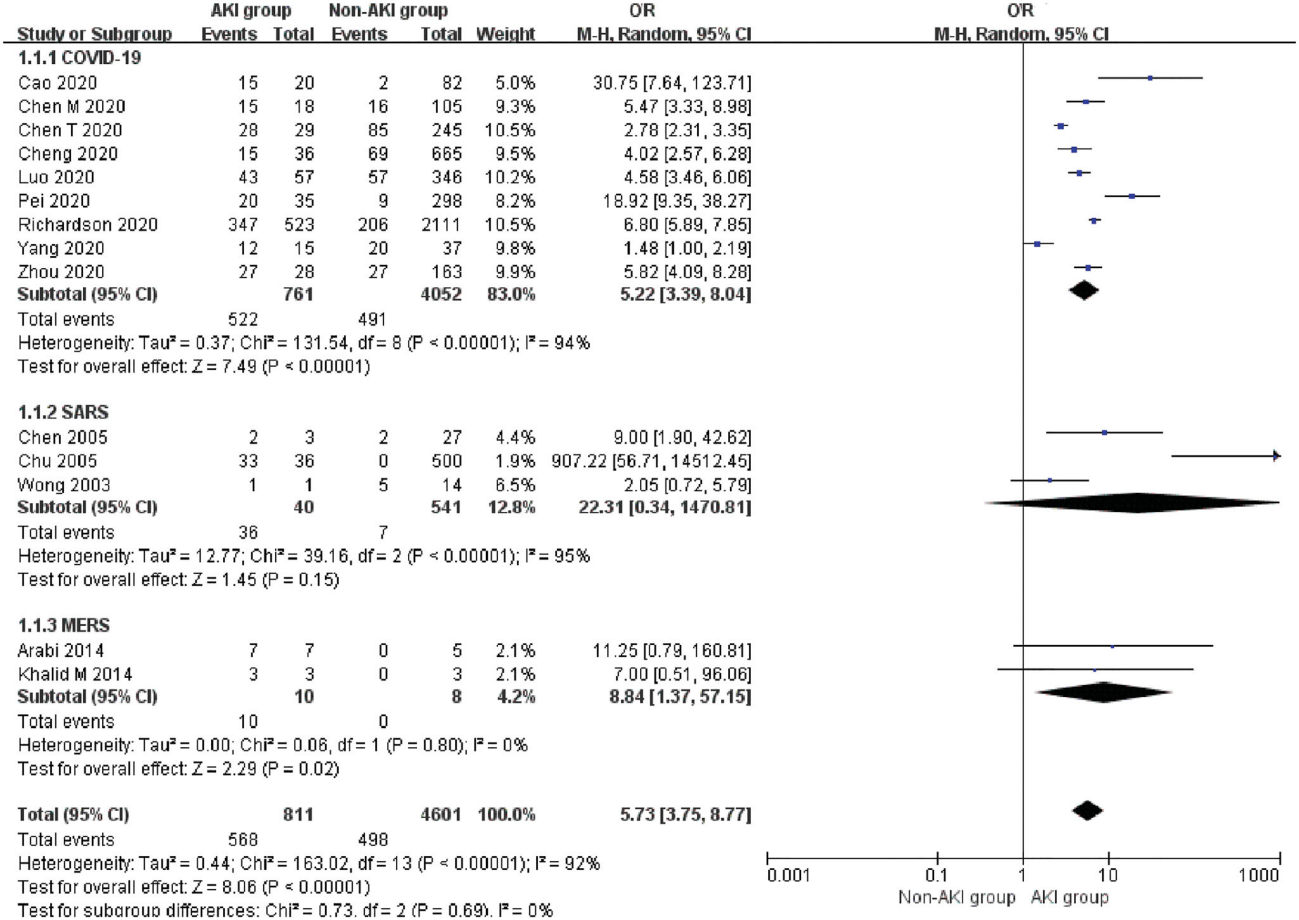
Mortality risk of AKI in three types of coronavirus diseases compared with non-AKI. AKI: acute kidney injury; SARS: severe acute respiratory syndrome; MERS: Middle East respiratory syndrome; COVID-19: novel coronavirus disease 2019.

The overall incidence of AKI was 12.5% (95% CI: 7.6%–18.3%, Heterogeneity *I*^2^ = 97.8%, *p* < 0.001; Supplementary Figure 1) adjusted for sample size with an overall mortality rate of 80.9% (95% CI: 57.6%–97.4%, Heterogeneity *I*^2^ = 96.1%, *p* < 0.001, Supplementary Figure 2). AKI significantly increased the risk of mortality (OR 5.73, 95% CI 3.75 to 8.77, *p* < 0.00001; *I*^2^ = 92%, *p* < 0.00001, Figure 2) in patients with coronavirus infection.

The incidence of AKI was 9.0% (95% CI: 4.2%–15.2%) in patients with COVID-19 but varied from 8.3% to 28.85% in the four studies reporting COVID-19 related AKI on ICU [81]. The mortality rate of AKI patients with COVID-19 was 72.3% (95% CI: 47.1%–92.0%) in nine studies [27–29,32–34,43,45,55]. AKI was associated with a higher risk of mortality compared with non-AKI patients (Nine studies [27–29,32–34,43,45,55], OR 5.22, 95% CI 3.39 to 8.04, *p* < 0.00001; *I*^2^ = 94%, *p* < 0.00001, Figure 2) in COVID-19 patients. *T*-statistic was performed to check the stability of this result: *t* = 6.015, *p* = 0.0003. There was no significant publication bias (Begg’s test: *p* = 0.251, and Egger’s test: *p* = 0.304). Funnel plot was nearly symmetrical (Supplementary Figure 3).

The incidence of AKI was 9.6% (95% CI: 3.9%–17.2%) in SARS patients. AKI occurred in 51 out of 641 SARS patients. The mortality rate of AKI patients with SARS was 98.9% (95% CI: 86.9%–100.0%) in three studies [56,57,59]. However, AKI itself was not associated with a significantly higher risk of mortality (three studies [56,57,60], OR 22.31, 95% CI 0.34 to 1470.81, *p* = 0.15; *I*^2^ = 95%, *p* < 0.00001, Figure 2) in these studies. *T*-statistic was performed to check the stability of this result: *t* = 1.459, *p* = 0.282.

The incidence of AKI was the highest in MERS, which was 42.0% (95% CI: 29.8%–54.7%). The mortality rate of AKI patients with MERS was 100.0% (95% CI: 82.4%–100.0%) based on two studies [3,72]. AKI was associated with a significantly higher risk of mortality (Two studies [3,72], OR 8.84, 95% CI 1.37 to 57.15, *p* = 0.02; *I*^2^ = 0%, *p* = 0.80, Figure 2) in MERS patients. However, *T*-statistic showed that *t* = 9.202 and *p* = 0.069.

### Urgent-start KRT application in patients with coronavirus infection

The overall rate of urgent-start KRT use was 8.9% (95% CI: 5.0%–13.8%, Heterogeneity *I*^2^ = 97.2%, *p* < 0.001, Supplementary Figure 4) but only one [56] study reported the use of urgent-start KRT in SARS patients with a rate of 1.87% and no deaths.

**Figure 3.**
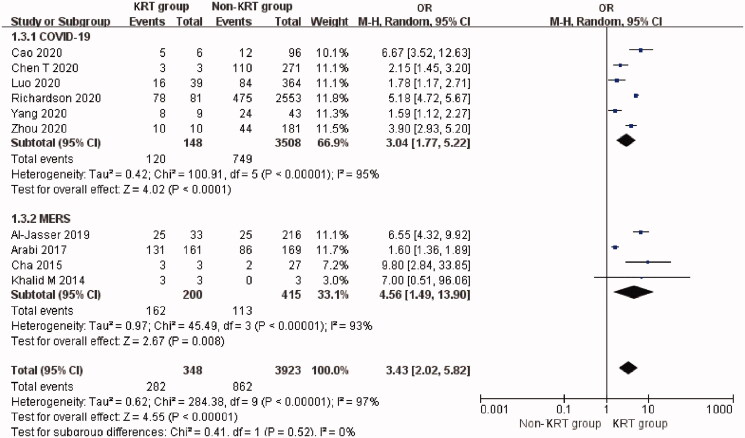
Mortality risk of urgent-start KRT use in three types of coronavirus diseases. Urgent-start KRT: urgent-start renal replacement therapy; MERS: Middle East respiratory syndrome; COVID-19: novel coronavirus disease 2019.

Urgent-start KRT-treated patients with coronavirus infection had an overall mortality of 80.7% (95% CI: 58.8%–96.6%, Heterogeneity *I*^2^ = 92.9%, *p* < 0.001, Supplementary Figure 5). The use of urgent-start KRT was significantly associated with increased mortality (OR 3.43, 95% CI 2.02 to 5.82, *p* < 0.00001; *I*^2^ = 97%, *p* < 0.00001, Figure 3) although this applied only to patients with MERS and COVID-19.

So far, 13 studies [6,26,27,29,31–35,39,40,45,55] have reported the rate of urgent-start KRT use (3.4%, 95% CI: 1.9%–5.4%) in hospitalized patients with COVID-19. The mortality rate of urgent-start KRT-treated patients with COVID-19 was 74.2% (95% CI: 45.8%–95.5%) in six studies [27,29,32–34,55]. The use of urgent-start KRT was associated with a higher risk of mortality compared with non-KRT patients (Six studies [27,29,32–34,55], OR 3.04, 95% CI 1.77 to 5.22, *p* < 0.0001; *I*^2^ = 95%, *p* < 0.00001, Figure 3) in COVID-19. *T*-statistic showed that *t* = 4.597 and *p* = 0.006. Sensitivity analysis by removal of Richardson *et al.*’s study [55] resulted in a 10% reduction of heterogeneity for mortality.

Seven studies [3,63,65,67,72,77,80] reported the highest rate of urgent-start KRT use (35.0%, 95% CI: 16.8%–55.4%) in hospitalized patients with MERS. The mortality rate of urgent-start KRT patients with MERS was 85.5% (95% CI: 78.9%–91.2%) in four studies [65,67,72,80]. The use of urgent-start KRT was also associated with a higher risk of mortality in MERS patients (Four studies [65,67,72,80], OR 4.56, 95% CI 1.49 to 13.90, *p* = 0.008; *I*^2^ = 93%, *p* < 0.00001, Figure 3). *T*-statistic showed that *t* = 3.365 and *p* = 0.044.

### Pre-dialysis CKD prevalence and mortality risk in patients with coronavirus infection

**Figure 4.**
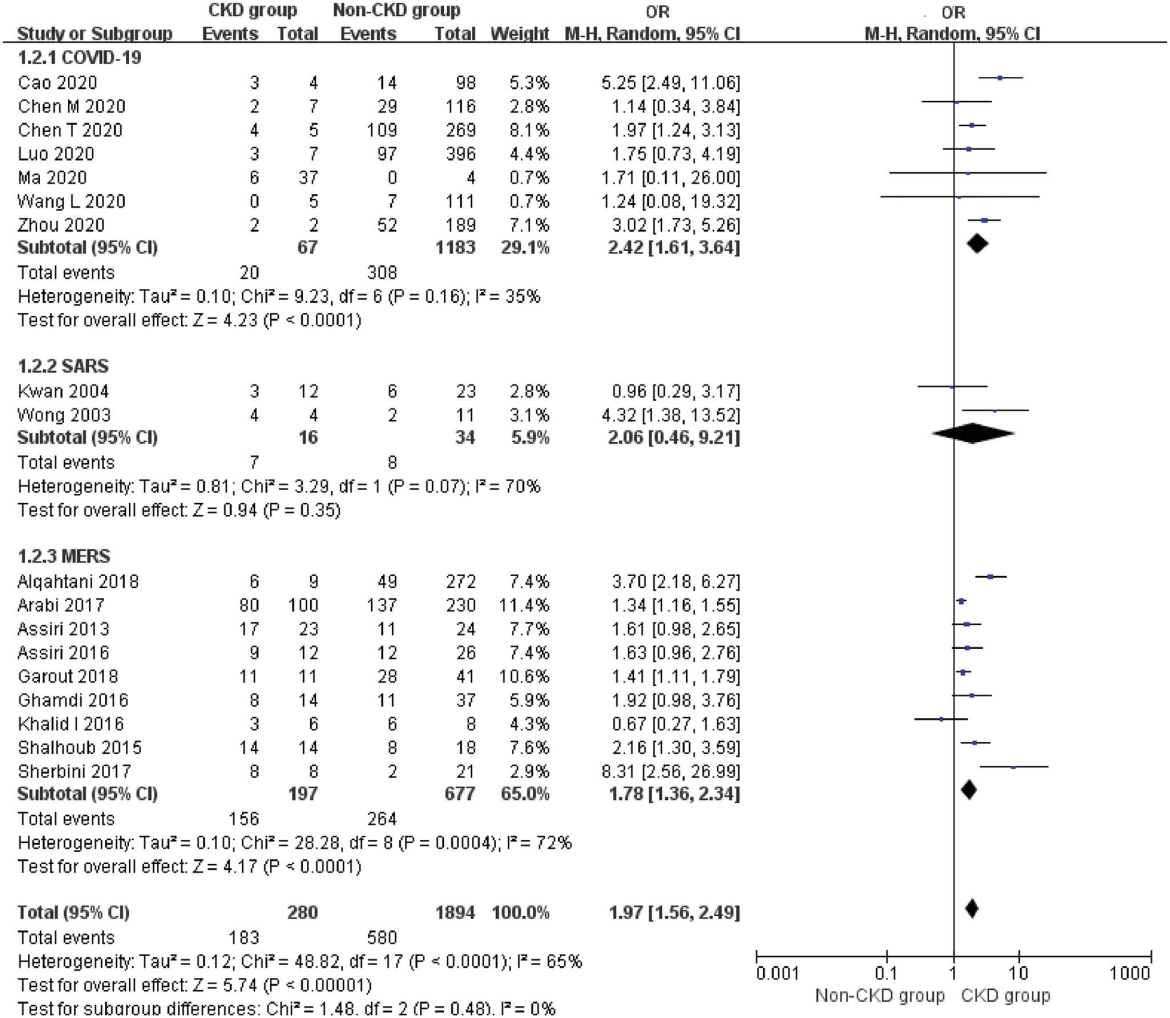
Mortality risk of non-dialytic preexisting CKD in three types of coronavirus diseases compared with non-CKD. CKD: chronic kidney disease; SARS: severe acute respiratory syndrome; MERS: Middle East respiratory syndrome; COVID-19: novel coronavirus disease 2019.

The overall prevalence of CKD was 14.2% (95% CI: 9.6%–19.6%, Heterogeneity *I*^2^ = 97.6%, *p* < 0.001, Supplementary Figure 6). The mortality rate of CKD patients with coronavirus infection was 65.4% (95% CI: 46.3%–82.7%, Heterogeneity *I*^2^ = 87.3%, *p* < 0.001, Supplementary Figure 7). CKD significantly increased the risk of mortality (OR 1.97, 95% CI 1.56 to 2.49, *p* < 0.00001; *I*^2^ = 65%, *p* < 0.0001, Figure 4) in patients with coronavirus infection.

The prevalence of CKD comorbidity was 11.0% (95% CI: 5.6%–17.8%) in COVID-19 patients with an associated mortality rate of 38.7% (95% CI: 16.8%–62.7%) in eight studies [27–29,33,34,37,38,51]. CKD was associated with a significantly higher risk of mortality compared with non-CKD patients with COVID-19 (Seven studies [27–29,33,34,37,38], OR 2.42, 95% CI 1.61 to 3.64, *p* < 0.0001; *I*^2^ = 35%, *p* = 0.16, Figure 4). *T*-statistic showed that *t* = 4.605 and *p* = 0.004. Sensitivity analysis by removal of a single-study showed that Cao *et al.*’s study [34] contributed about 22% of the heterogeneity.

The prevalence of CKD was 4.4% (95% CI: 0.0%–19.0%) in SARS patients. The mortality rate of CKD patients with SARS was 46.5% (95% CI: 20.6%–73.2%) in two studies [57,61]. CKD was not associated with a significantly higher risk of mortality in SARS patients although this analysis was based on only two studies (Two studies [57,61], OR 2.06, 95% CI 0.46 to 9.21, *p* = 0.35; *I*^2^ = 70%, *p* = 0.07, Figure 4). *T*-statistic showed that *t* = 0.998 and *p* = 0.501.

The prevalence of CKD was 23.8% (95% CI: 15.8%–32.7%) in MERS patients. The mortality rate of CKD patients with MERS was very high, i.e. 83.6% (95% CI: 69.4%–94.7%). As for the nine articles [63,65,66,68,69,73,74,76,79] describing the prognosis of 197 CKD patients versus 677 non-CKD patients with MERS, pooled analysis of the mortality revealed a significantly higher risk of mortality in MERS patients with CKD (OR 1.78, 95% CI 1.36 to 2.34, *p* < 0.0001; *I*^2^ = 72%, *p* = 0.0004, Figure 4). *T*-statistic showed that *t* = 3.244 and *p* = 0.012. There was no significant publication bias (Begg’s test: *p* = 0.118, and Egger’s test: *p* = 0.075) but the Funnel plot was not so symmetrical (Supplementary Figure 8).

### ESKD prevalence and mortality risk in patients with coronavirus infection

**Figure 5.**
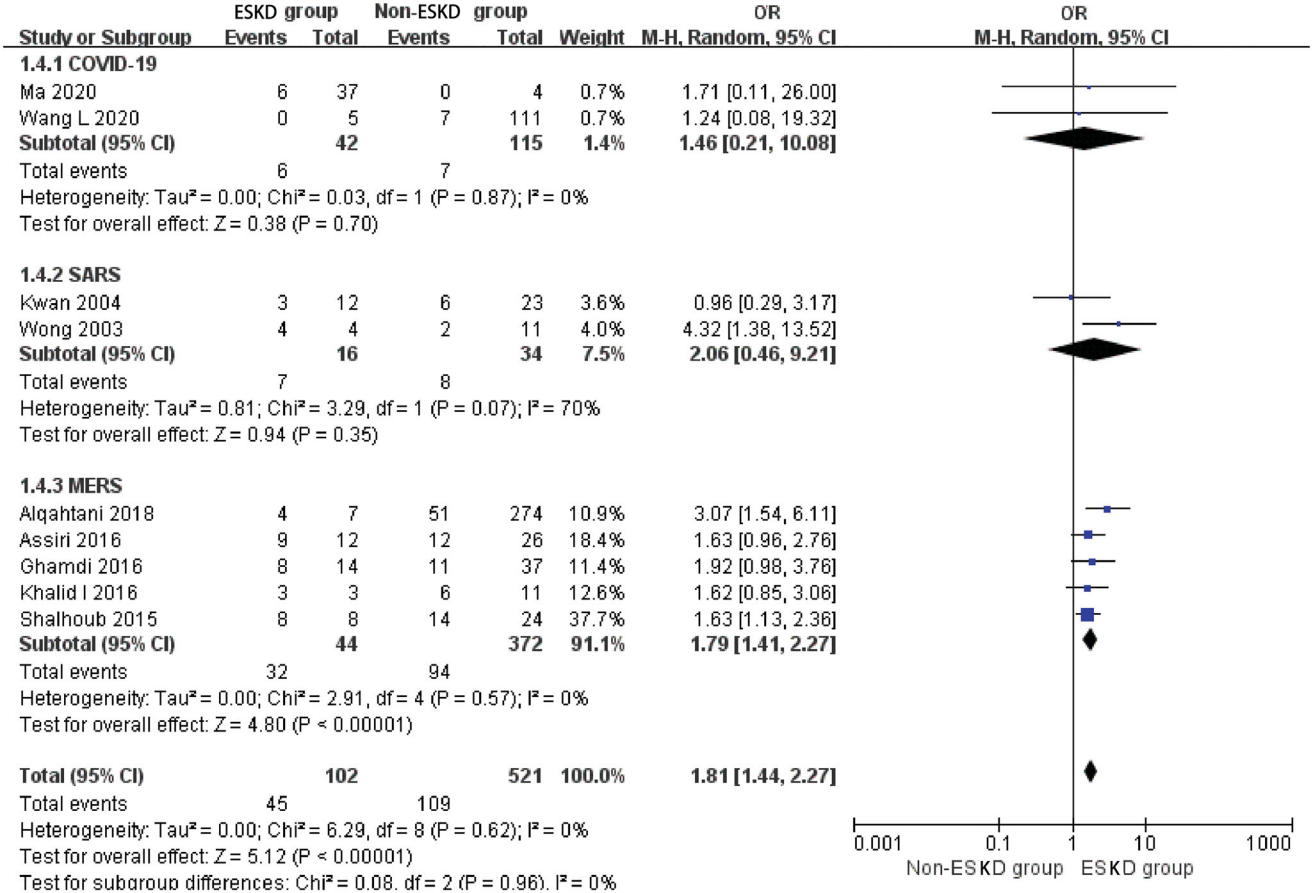
Mortality risk of preexisting ESKD in three types of coronavirus diseases compared with non-ESKD. ESKD: end-stage renal disease; SARS: severe acute respiratory syndrome; MERS: Middle East respiratory syndrome; COVID-19: novel coronavirus disease 2019.

The overall prevalence of ESKD was 16.4% (95% CI: 7.2%–27.9%, Heterogeneity *I*^2^ = 98.2%, *p* < 0.001, Supplementary Figure 9). The overall mortality rate of ESKD patients with coronavirus infection was 51.7% (95% CI: 27.0%–76.1%, Heterogeneity *I*^2^ = 83.3%, *p* < 0.001, Supplementary Figure 10). Overall analysis showed that ESKD significantly increased the risk of mortality (OR 1.81, 95% CI 1.44 to 2.27, *p* < 0.00001; *I*^2^ = 0%, *p* = 0.62, Figure 5) in patients with coronavirus infection.

The prevalence of ESKD was 30.9% (95% CI: 4.6%–66.8%) in the COVID-19-related studies. The mortality rate of ESKD patients with COVID-19 was 17.6% (95% CI: 8.2%–29.2%). Compared with non-ESKD patients, ESKD was not associated with a higher risk of mortality although this was based on two studies (two studies [37,38], OR 1.46, 95% CI 0.21 to 10.08, *p* = 0.70; *I*^2^ = 0%, *p* = 0.87, Figure 5) in SARS-CoV-2. *T*-statistic showed that *t* = 2.339 and *p* = 0.257.

The prevalence of ESKD was 4.4% (95% CI: 0.0%–19.0%) in SARS related studies. The mortality rate of ESKD patients with SARS was 46.5% (95% CI: 20.6%–73.2%). ESKD was also not associated with a higher risk of mortality (Two studies [57,61], OR 2.06, 95% CI 0.46 to 9.21, *p* = 0.35; *I*^2^ = 70%, *p* = 0.07, Figure 5) in SARS patients. *T*-statistic showed that *t* = 0.999 and *p* = 0.500.

The prevalence of ESKD was 13.8% (95% CI: 5.1%–25.2%) included MERS related studies. The mortality rate of ESKD patients with MERS was the highest: 78.1% (95% CI: 51.1%–97.6%). The pooled analysis of the mortality revealed a significantly higher risk of mortality in MERS patients with ESKD (Five studies [63,68,74,76,79], OR 1.79, 95% CI 1.41 to 2.27, *p* < 0.00001; *I*^2^ = 0%, *p* = 0.57, Figure 5). *T*-statistic showed that *t* = 5.682 and *p* = 0.005.

### Patients on chronic hemodialysis and the occurrence of coronavirus infection

The overall incidence of coronavirus infection was 7.7% (95% CI: 4.9%–11.1%, Heterogeneity *I*^2^ = 97.2%, *p* < 0.001, Supplementary Figure 11) with a mortality rate of 26.4% (95% CI: 20.6%–32.6%, Heterogeneity *I*^2^ = 51.6%, *p* < 0.001, Supplementary Figure 12).

The incidence of COVID-19 was 8.0% (nine studies [30,38,42,44,46–50], 95% CI: 4.7%–12.0%) in hemodialysis patients with a mortality rate of 25.7% (nine studies [30,38,42,44,46,49–51,53], 95% CI: 21.3%–30.3%). The incidence of SARS was 1.7% (95% CI: 0.9%–3.0%) based on a single study [61] with a mortality rate of 25.0% (95% CI: 5.5%–57.2%). The incidence of MERS was 3.6% (95% CI: 1.8%–5.9%) from two studies [74,75] in hemodialysis patients with an associated mortality rate of 75.0% (one study [74], 95% CI: 42.8%–94.5%).

### Sensitivity analysis and meta-regression analysis

We further conducted sensitivity analysis to evaluate the influence of case series and preprinted literatures on the stability of results. First, the results maintained significance after excluding all the preprinted literatures included in the pooled analysis [27,28,38,39,41]. Second, the results also maintained stable by excluding the literatures included in the pooled analysis one by one. Moreover, too few studies were left in each subgroup after excluding all the case series, because this type of study occupied a relatively large proportion (about 50%). Thus, we kept the case series with number of patients reported equal or greater than 5 cases, and rated the quality of these literatures referring to a generally recommended standard [17]. Meta-regression analysis was used to find potential heterogeneity in primary results. However, different ethnicities and study types did not contribute significantly to the heterogeneity in four results (P all > 0.05).

## Discussion

The COVID-19 pandemic that is currently raging around the world is causing major disruption to health systems [82]. As a member of the coronavirus family [2], COVID-19 together with SARS and MERS lead to severe acute respiratory symptoms [83], as well as extrapulmonary disease [84]. Although the kidney is commonly affected, its contribution to patient mortality and morbidity is only belatedly being recognized. Compared with similar systematic reviews [85,86] that had been published so far, our research explored the impact of kidney-related events on the prognosis of patients in the face of coronavirus abuse from a more comprehensive and in-depth perspective. We conducted this systematic review to investigate the incidence of AKI, the increased risk to patients with preexisting CKD, ESKD or urgent-start KRT and differences in kidney outcomes for all three recent coronavirus pandemics.

Our results indicate that AKI occurs in around one-tenth of the infected study population with an overall mortality rate of 80.9%. The incidence of AKI was highest in MERS patients, while being similar between COVID-19 and SARS patients. The incidence in ICU-treated patients varied between 8.3% and 28.85% [81]. Compared to COVID-19 patients, the mortality rate was higher in SARS and MERS patients although fewer studies were reported for the SARS [56,57,60] and MERS [3,72] subgroups. AKI was associated with a significantly higher mortality in COVID-19 and MERS. In the SARS subgroup, this did not reach statistical significance possibly due to the small number of studies included. The incidence of urgent-start KRT use in coronavirus infected patients with AKI was 8.9% with an associated mortality of 80.7%. This probably reflects the fact that AKI patients requiring urgent-start KRT are generally more critically ill, likely to need ventilatory support or extracorporeal membrane oxygenation (ECMO) [87]. Many dialysis modalities [88–90], including CRRT, high-volume hemofiltration, plasma exchange, plasma adsorption and acute peritoneal dialysis have been reported, mostly as case reports or small case series. A consensus recommendation regarding the optimal dialysis modality, timing, dosage and duration for management of AKI in coronavirus diseases is urgently needed.

Our analysis also showed that the presence of CKD or ESKD was significantly associated with increased mortality. The overall prevalence of ESKD was higher than that of preexisting CKD, possibly due to different number of studies being enrolled for each analysis. Patients with MERS had the highest mortality in prevalent patients with CKD or ESKD. Several studies have reported on virus prevalence and mortality in patients on prevalent hemodialysis [30,38,42,44,46–51,53,54,61,74,75]. The incidence of COVID-19 was 8.0% in routine hemodialysis patients, which was higher than SARS or MERS, but similar to the general population. The mortality rate for this subgroup was 25.7%, which was nearly the same as SARS but much less than with MERS (75%). Our analysis confirms that prevalent patients with CKD or on urgent-start KRT are at much higher risk of infection and of subsequent worse outcomes. Epidemic prevention measures must be strengthened especially in dialysis centers [91]. Specific measures that could be introduced include the setting up of isolation areas for dialysis centers, wearing personal protective equipment, tracking and isolating contacts and environmental disinfection. For infected patients, continuous bedside dialysis has been successfully deployed [92].

Our study had several limitations. First, we combined studies with a certain degree of heterogeneity, owing to the differences in the study design, sample size and population characteristics of the studies included. The specific reasons were as follows: (1) inclusion of case control study and case series can introduce bias to the result, which may lead to the high heterogeneity; (2) the number of infected patients enrolled in the included articles varied widely; (3) the inconsistent definitions of AKI or CKD or ESKD could have accounted for the variation of our results on AKI; (4) differences in the timing of outbreaks, geographical locations, ages, genders, habits, cares, and treatments may also contribute to the high heterogeneity; (5) the total sample size of SARS and MERS related studies was much smaller than COVID-19. Second, sampling bias may have contributed to part of our analysis when less than five cases were excluded. Third, renal function follow-up to assess renal recovery was not available. Fourth, several case series (about 8.62%) were included in our study, which could reduce the strength of the generated evidences. Then we tried to do the sensitivity analysis excluding all the case series, but found too few studies left. Also shown with the GRADE tool, the level of evidences generated in this study were low or very low; thus, more future high-quality researches are urged to confirm our results.

In conclusion, the kidney is commonly affectedly in patients with COVID-19, SARS and MERS. Renal events including AKI, preexisting CKD, and ESKD significantly increased the risk of mortality. Prevalent patients on urgent-start KRT also have an increased risk of infection and mortality. Routine hemodialysis patients were also at high risk of infection and mortality.

## Supplementary Material

Supplemental Material Table 1

Supplemental Material Table 2
