# Supplementary material for: Coronavirus-associated kidney outcomes in COVID-19, SARS, and MERS: a meta-analysis and systematic review
Source: Ren Fail. 2020 Nov 30;43(1):1–15. doi: 10.1080/0886022X.2020.1847724 (PMC7717867; doi:10.1080/0886022X.2020.1847724)
Supplement: Supplemental Material Table 1 [file IRNF_A_1847724_SM0462.docx]

**Supplementary table 1**. Assessment of the quality of the included studies by NOS.

| Studies | Selection | | | |  | Comparability |  | Outcome assessment | | | NOS score |
| --- | --- | --- | --- | --- | --- | --- | --- | --- | --- | --- | --- |
|  | ① | ② | ③ | ④ |  | ⑤ |  | ⑥ | ⑦ | ⑧ |  |
| Wong 2003 | c | a | a | a |  | c |  | a | a | a | 6 |
| Gu 2005 | c | c | a | b |  | c |  | a | a | a | 4 |
| Chen 2005 | b | a | a | a |  | c |  | a | a | a | 7 |
| Wu 2005 | b | a | a | a |  | a |  | a | a | a | 8 |
| Farcas 2005 | c | c | a | b |  | c |  | a | a | a | 4 |
| Chu 2005 | b | a | a | b |  | c |  | a | a | a | 6 |
| Kwan 2004 | c | a | a | b |  | a |  | a | a | a | 5 |
| Peiris 2003 | b | c | a | a |  | c |  | a | a | a | 6 |
| Al-Jasser 2019 | b | a | a | b |  | c |  | a | a | a | 6 |
| Assiri 2016 | b | a | a | b |  | c |  | a | a | a | 6 |
| Garout 2018 | c | a | a | b |  | c |  | a | a | a | 5 |
| Sherbini 2017 | b | a | a | b |  | c |  | a | a | a | 6 |
| Shalhoub 2015 | b | a | a | b |  | c |  | a | a | a | 6 |
| Hastings 2016 | b | a | a | b |  | c |  | a | a | a | 6 |
| Arabi 2017 | a | a | a | b |  | c |  | a | a | a | 6 |
| Assiri 2013 | b | a | a | a |  | c |  | a | a | a | 7 |
| Alfaraj 2019 (1) | b | a | a | a |  | c |  | a | a | a | 7 |
| Alqahtani 2018 | b | a | a | b |  | c |  | a | a | a | 6 |
| Alanazi 2019 | b | a | a | a |  | c |  | a | a | a | 7 |
| Alfaraj 2019 (2) | b | c | a | b |  | c |  | a | a | a | 5 |
| Al-Tawfiq 2014 | b | a | a | b |  | ab |  | a | a | a | 8 |
| Arabi 2014 | c | a | a | a |  | c |  | a | a | a | 6 |
| Cha 2015 | b | a | a | a |  | a |  | a | a | a | 8 |
| Ghamdi 2016 | b | a | a | b |  | c |  | a | a | a | 6 |
| Khalid I 2016 | b | a | a | b |  | c |  | a | a | a | 6 |
| Khalid M 2014 | c | c | a | a |  | c |  | a | a | a | 5 |
| Omrani 2014 | b | a | a | b |  | c |  | a | a | a | 6 |
| Ma 2020 | c | a | a | a |  | c |  | a | b | a | 5 |
| Richardson 2020 | b | a | a | a |  | c |  | a | b | a | 6 |
| Chen N 2020 | b | a | a | b |  | c |  | a | b | a | 5 |
| Lu 2020 | b | a | a | b |  | c |  | a | b | a | 5 |
| Wang D 2020 | b | a | a | b |  | c |  | a | b | a | 5 |
| Chen T 2020 | b | a | a | b |  | c |  | a | a | a | 6 |
| Xu 2020 | b | a | a | b |  | c |  | a | b | a | 5 |
| Huang 2020 | b | a | a | a |  | c |  | a | b | a | 6 |
| Diao 2020 | b | a | a | a |  | c |  | d | b | d | 4 |
| Cao 2020 | b | a | a | b |  | c |  | a | a | a | 6 |
| Arentz 2020 | c | a | a | a |  | c |  | a | b | a | 5 |
| Cheng 2020 | b | a | a | a |  | a |  | a | a | a | 8 |
| Guan 2020 | b | c | a | b |  | c |  | a | a | a | 5 |
| Shi 2020 | b | c | a | b |  | c |  | a | b | a | 4 |
| Wang L 2020 | b | a | a | a |  | c |  | a | a | a | 7 |
| Yang 2020 | b | a | a | b |  | c |  | a | a | a | 6 |
| Pei 2020 | b | a | a | b |  | ab |  | a | a | a | 8 |
| Xiong 2020 | c | a | a | b |  | c |  | a | a | a | 5 |
| Luo 2020 | b | a | a | b |  | c |  | a | a | a | 6 |
| Zhou 2020 | b | a | a | b |  | c |  | a | a | a | 6 |
| Albalate 2020 | c | c | a | a |  | c |  | a | a | a | 5 |
| Valeri 2020 | c | c | a | b |  | c |  | a | a | a | 4 |
| Chen M 2020 | b | a | a | b |  | c |  | a | a | a | 6 |
| Jung 2020 | c | c | a | b |  | c |  | a | a | a | 4 |
| Arslan 2020 | c | c | a | b |  | c |  | a | a | a | 4 |
| Alberici 2020 | c | c | a | a |  | c |  | a | a | a | 5 |
| Goicoechea 2020 | c | c | a | b |  | c |  | a | a | a | 4 |
| Dudreuilh 2020 | c | c | d | b |  | c |  | a | a | a | 3 |
| Trujillo 2020 | c | c | a | b |  | c |  | a | a | a | 4 |
| Manganaro 2020 | b | a | a | b |  | c |  | a | a | a | 6 |
| Fisher 2020 | c | c | a | b |  | c |  | a | b | b | 3 |

① Representativeness of the exposed cohort; ② selection of the non-exposed cohort; ③ ascertainment of exposure; ④ demonstration that outcome of interest was not present at start of study; ⑤ comparability of cohorts on the basis of the design or analysis; ⑥ assessment of outcome; ⑦ was follow-up long enough for outcomes to occur; ⑧ adequacy of follow up of cohorts.

**NEWCASTLE - OTTAWA QUALITY ASSESSMENT SCALE**

**COHORT STUDIES**

Note: A study can be awarded a maximum of one star for each numbered item within the Selection and Outcome categories. A maximum of two stars can be given for Comparability

**Selection**

1) Representativeness of the exposed cohort

a) truly representative of the average _______________ (describe) in the community **🟑**

b) somewhat representative of the average ______________ in the community **🟑**

c) selected group of users eg nurses, volunteers

d) no description of the derivation of the cohort

2) Selection of the non exposed cohort

a) drawn from the same community as the exposed cohort **🟑**

b) drawn from a different source

c) no description of the derivation of the non exposed cohort

3) Ascertainment of exposure

a) secure record (eg surgical records) **🟑**

b) structured interview **🟑**

c) written self report

d) no description

4) Demonstration that outcome of interest was not present at start of study

a) yes **🟑**

b) no

**Comparability**

1) Comparability of cohorts on the basis of the design or analysis

a) study controls for _____________ (select the most important factor) **🟑**

b) study controls for any additional factor **🟑** (This criteria could be modified to indicate specific control for a second important factor.)

**Outcome**

1) Assessment of outcome

a) independent blind assessment **🟑**

b) record linkage **🟑**

c) self report

d) no description

2) Was follow-up long enough for outcomes to occur

a) yes (select an adequate follow up period for outcome of interest) **🟑**

b) no

3) Adequacy of follow up of cohorts

a) complete follow up - all subjects accounted for **🟑**

b) subjects lost to follow up unlikely to introduce bias - small number lost - > ____ % (select an adequate %) follow up, or description provided of those lost) **🟑**

c) follow up rate < ____% (select an adequate %) and no description of those lost

d) no statement.
