# Supplementary material for: Coronavirus-associated kidney outcomes in COVID-19, SARS, and MERS: a meta-analysis and systematic review
Source: Ren Fail. 2020 Nov 30;43(1):1–15. doi: 10.1080/0886022X.2020.1847724 (PMC7717867; doi:10.1080/0886022X.2020.1847724)
Supplement: Supplemental Material Table 2 [file IRNF_A_1847724_SM0449.docx]

**Supplementary Table 2**. Summary of Findings Table: The four most important patient outcomes are listed in the summary of findings table

| **Outcomes** | **Relative effect (95% CI)** | **No of Participants (studies)** | **Quality of the evidence (GRADE)** |
| --- | --- | --- | --- |
|  |  |  |  |
|  |  |  |  |
| **AKI vs Non-AKI group mortality risk** | **RR 5.73**  (3.75 to 8.77) | 5412 (14 studies^1^) | ⊕⊝⊝⊝ **very low**^1^ |
| **AKI vs Non-AKI group mortality risk- COVID-19 subgroup** | **RR 5.22**  (3.39 to 8.04) | 4813 (9 studies^1^) | ⊕⊝⊝⊝ **very low**^1^ |
| **AKI vs Non-AKI group mortality risk- SARS subgroup** | **RR 22.31**  (0.34 to 1470.81) | 581 (3 studies^1^) | ⊕⊝⊝⊝ **very low**^1^ |
| **AKI vs Non-AKI group mortality risk- MERS subgroup** | **RR 8.84**  (1.37 to 57.15) | 18 (2 studies^1^) | ⊕⊝⊝⊝ **very low**^1,2^ |
| **Urgent-start RRT vs Non-RRT application mortality risk** | **RR 3.43**  (2.02 to 5.82) | 4271 (10 studies^1^) | ⊕⊝⊝⊝ **very low**^1,3^ |
| **Urgent-start RRT vs Non-RRT application mortality risk - COVID-19 subgroup** | **RR 3.04**  (1.77 to 5.22) | 3656 (6 studies^1^) | ⊕⊕⊝⊝ **low**^1,3^ |
| **Urgent-start RRT vs Non-RRT application mortality risk - MERS subgroup** | **RR 4.56**  (1.49 to 13.9) | 615 (4 studies^1^) | ⊕⊝⊝⊝ **very low**^1^ |
| **Pre-dialysis CKD vs Non-CKD group mortality risk** | **RR 1.97**  (1.56 to 2.49) | 2174 (18 studies^1^) | ⊕⊝⊝⊝ **very low**^1^ |
| **Pre-dialysis CKD vs Non-CKD group mortality risk - COVID-19 subgroup** | **RR 2.42**  (1.61 to 3.64) | 1250 (7 studies^1^) | ⊕⊕⊝⊝ **low** |
| **Pre-dialysis CKD vs Non-CKD group mortality risk - SARS subgroup** | Not estimable | 50 (2 studies^2^) | ⊕⊝⊝⊝ **very low**^2^ |
| **Pre-dialysis CKD vs Non-CKD group mortality risk - MERS subgroup** | **RR 1.78**  (1.36 to 2.34) | 874 (9 studies^1^) | ⊕⊕⊝⊝ **low** |
| **ESRD vs Non-ESRD group mortality risk** | **RR 1.81**  (1.44 to 2.27) | 623 (9 studies^1^) | ⊕⊝⊝⊝ **very low**^1^ |
| **ESRD vs Non-ESRD group mortality risk - COVID-19 subgroup** | Not estimable | 157 (2 studies^2^) | ⊕⊝⊝⊝ **very low**^2^ |
| **ESRD vs Non-ESRD group mortality risk - SARS subgroup** | Not estimable | 50 (2 studies^2^) | ⊕⊝⊝⊝ **very low**^2^ |
| **ESRD vs Non-ESRD group mortality risk - MERS subgroup** | **RR 1.79**  (1.41 to 2.27) | 416 (5 studies^1^) | ⊕⊝⊝⊝ **very low**^1^ |
| ***The basis for the assumed risk (e.g. the median control group risk across studies) is provided in footnotes. The corresponding risk (and its 95% confidence interval) is based on the assumed risk in the comparison group and the relative effect of the intervention (and its 95% CI).  CI: Confidence interval; RR: Risk ratio;** | | | |
| **GRADE Working Group grades of evidence High quality: Further research is very unlikely to change our confidence in the estimate of effect.  Moderate quality: Further research is likely to have an important impact on our confidence in the estimate of effect and may change the estimate. Low quality: Further research is very likely to have an important impact on our confidence in the estimate of effect and is likely to change the estimate. Very low quality: We are very uncertain about the estimate.** | | | |
| **1 case-control and other study designs together 2 case series 3 case-control** | | | |
